# Novel Cause of Tuberculosis in Meerkats, South Africa

**DOI:** 10.3201/eid1912.130268

**Published:** 2013-12

**Authors:** Sven D.C. Parsons, Julian A. Drewe, Nicolaas C. Gey van Pittius, Robin M. Warren, Paul D. van Helden

**Affiliations:** Stellenbosch University, Tygerberg, South Africa (S.D.C. Parsons, N.C. Gey van Pittius, R.M. Warren, P.D. van Helden);; Royal Veterinary College, London, UK (J.A. Drewe);; South African Medical Research Council, Cape Town, South Africa (R.M. Warren, P.D. van Helden)

**Keywords:** meerkat, *Mycobacterium suricattae*, suricate, tuberculosis, South Africa, tuberculosis and other mycobacteria

## Abstract

The organism that causes tuberculosis in meerkats (*Suricata suricatta*) has been poorly characterized. Our genetic analysis showed it to be a novel member of the *Mycobacterium tuberculosis* complex and closely related to the dassie bacillus. We have named this epidemiologically and genetically unique strain *M. suricattae*.

Tuberculosis (TB) is caused by a group of distinct mycobacterial strains that might have evolved as host-adapted ecotypes (*1*) and that are collectively named the *Mycobacterium tuberculosis* complex (MTC) (*2*). In southern Africa, *M. tuberculosis* and *M. bovis* cause TB in numerous animals (*3*), the dassie bacillus infects rock hyraxes (dassies, *Procavia capensis*), and *M. mungi* infects banded mongooses (*Mungos mungo*) (*4*–*6*).

TB in free-living meerkats (*Suricata suricatta*) from the Kalahari Desert, South Africa, was first reported in 2002 (*7*), and its epidemiology and pathology have been comprehensively described (*8*,*9*). Mycobacterial strains isolated from these animals have been described as *M. tuberculosis* (*7*), *M. bovis* (*8*), and as a “member of the animal-adapted lineage of the MTC” (*10*), raising concerns that the occurrence of TB in these meerkats resulted from anthropogenic exposure to these pathogens and that affected meerkat populations could pose an infection risk to other wildlife, domestic animals, and humans (*8*). However, these studies used genetic analyses designed to differentiate between *M. tuberculosis* and *M. bovis* (*7*) and between these and *M. africanum*, *M. canetti*, *M. microti*, and *M. bovis* BCG (*8*) but not between these strains and the dassie bacillus or *M. mungi*. To gain greater insight into the etiology of this disease, we conducted a more comprehensive genetic analysis of mycobacterial isolates from this meerkat population.

## The Study

Permission to sample meerkats was obtained from the University of Pretoria Animal Ethics Committee. Postmortem examinations were performed on 4 meerkats from the Kalahari Meerkat Project (26°58′S, 21°49′E) that had shown visible disease. Samples from lesions typical of TB in this species (*8*) were used to establish mycobacterial cultures in the BD BACTEC MGIT 960 Mycobacterial Detection System (Becton Dickinson, Franklin Lakes, NJ, USA) (*11*). Four cultures originating from 3 animals were positive by Ziehl-Neelsen stain and were grown further on Difco Middlebrook 7H10 Agar supplemented with 10% OADC Enrichment (Becton Dickinson) for 6–8 weeks, after which DNA was extracted (*11*). However, only 1 isolate (MK172) yielded sufficient DNA for DNA fingerprinting by the IS*6110* method (*12*). PCRs were conducted by using either heat-killed liquid cultures or purified DNA as a template.

Isolates were screened for the presence or absence of 4 phylogenetically informative genomic regions of difference (RDs) (*11*), and all showed deletion of RD9 but not of RD1, RD4, and RD12. This genotype is shared by *M. africanum*, *M. orygis*, and the dassie bacillus (*2*,*11*); isolates were therefore analyzed for the presence or absence of RD1^das^, a genetic marker specific for the dassie bacillus (*5*). Because this RD was deleted in all isolates, these were subsequently analyzed for the presence or absence of N-RD25^das^, RD5^das^, and RDVirS^das^ (*5*); a G→A single-nucleotide polymorphism (SNP) in Rv1510 (Rv1510^1129^); and a single-nucleotide deletion in Rv0911 (Rv0911^389^) (*2*). For all isolates, N-RD25^das^, RD5^das^, and RDVirS^das^ were deleted and Rv1510^1129^ and Rv0911^389^ were present, consistent with the dassie bacillus genotype (*2*,*5*). However, although the RD5^das^ deletion in this bacillus has been caused by the insertion of an inverted IS*6110* sequence (*5*), for the meerkat strain, sequencing of the RD5^das^ PCR product showed this region to be occupied by an IS*6110* sequence in a forward orientation, followed by a proline-proline-glutamate gene homologue.

Spoligotyping was performed according to the internationally standardized method (*13*). However, we repeatedly obtained no amplification of any spacer included in this array. We investigated the possible deletion of the direct-repeat region, the genomic region analyzed by spoligotyping, by attempting to amplify by PCR selected genetic sequences upstream and downstream thereof (Table 1). This analysis confirmed that much, if not all, of the direct-repeat region had been deleted in these isolates, together with ≈3,500 bp upstream and up to 1,700 bp downstream of this region (Table 1).

**Table 1:** PCR analysis of the genomic regions flanking the direct-repeat region of *Mycobacterium suricattae*

PCR target, bp	Forward primer, 5′→3′	Reverse primer, 5′ →3′	PCR result*
10–200†	TACCTACGCCACCACCTCAAG	TCAGTCTGCCGTGACTTCGG	–
966–1,518†	CCCTATGTGGATGCGTGGTTG	GGGTTTCGGGTTTGGCTTTCG	–
2,214–2,377†	GTGTCGCTGGCTGAGACC	GCTCCTTTCCATTTGCTGTC	–
3,506–3,730†	ACCGATAATCGCTTGACACC	CCCTCGTTCTCTAGCAGCAG	+
60–262‡	ACGTAACTGCCGCAACACCTC	AATATACGACATCAGCGACAA	–
335–906‡	CGGCTGCGAGTGGGCATTTAG	TCCCTGGCGGAGTTGAACGG	–
1,702–1,931‡	TATCTCCGGCTCTCTTTCCA	TCTTTAAGGACACCGCGTTC	+
2,603–2,763‡	GTTCCGATAGGCGAGAACAG	CCAGTTCGGGAAGGTAGTCA	+

*–, no product; +, successful amplification. †Upstream of *M. tuberculosis* H37Rv direct-repeat region. ‡Downstream of *M. tuberculosis* H37Rv direct-repeat region.

**Figure 1:**
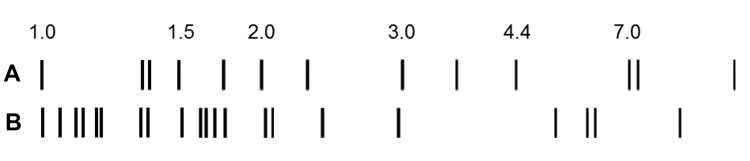
IS*6110* restriction fragment length polymorphism patterns of A) a reference strain of *Mycobacterium tuberculosis* (Mt14323) with selected fragment lengths indicated in kilobases, and B) *M. suricattae* (isolate MK172).

Additionally, genetic characterization was done by sequencing of fragments of the *gyrB* gene (*2*) and 16S rDNA (*14*). For all isolates, the *gyrB* sequence was consistent with that of *M. africanum*, *M. pinnipedii*, and the dassie bacillus (*2*). However, the 16S rDNA sequence differed from that of all other MTC members by having a T→G SNP at position 214 (16S rDNA^214^). Analysis by mycobacterial interspersed repetitive unit–variable number tandem repeats (*15*) identified 2 strain variants in our sample set (Table 2); IS*6110* DNA fingerprint analysis (*12*) of isolate MK172 showed it to contain 21 copies of the IS*6110* insertion sequence element (Figure 1).

**Table 2:** MIRU-VNTR patterns of *Mycobacterium suricattae* and representative isolates of selected members of the *M. tuberculosis* complex*

Locus	MIRU-VNTR copy number			
	*M. africanum*†	*M. mungi*†	*M. suricattae*	Dassie bacillus†
MIRU 2	2	2	2	2
VNTR 424/Mtub04	4	3	3‡, 2§	2
VNTR 577/ETR-C	5	3	5	5
MIRU 4/ETR-D	2	3	2	3
MIRU 40	2	1	2	2
MIRU 10	7	5	6	7
MIRU 16	4	3	2	3
VNTR 1955/Mtub21	4	3	3	3
MIRU 20	2	2	2	2
VNTR 2163b/QUB11b	5	–	–	7
VNTR 2165/ETR-A	6	6	–	6
VNTR2347/Mtub29	3	3	3	3
VNTR 2401/Mtub30	4	4	4‡, 5§	3
VNTR 2461/ETR-B	4	4	5	4
MIRU 23	4	4	4	4
MIRU 24	2	2	3	2
MIRU 26	4	4	4	5
MIRU 27	3	3	1	4
VNTR 3171/Mtub 34	3	3	3	3
MIRU 31/ETR-E	5	8 and 9	5	5
VNTR 3690/Mtub 39	4	–	8	5
VNTR 4052/QUB 26	6	–	3	4
VNTR 4156/QUB 4156	3	–	1	3
MIRU 39	2	2	2	2

*MIRU-VNTR, mycobacterial interspersed repetitive unit–variable number tandem repeats; –, no amplification. †From Alexander et al., 2010 (*4*).  ‡Copy number of 3 isolates from 2 meerkats, including MK172.  §Copy number of a fourth isolate from a third meerkat.

## Conclusion

**Figure 2:**
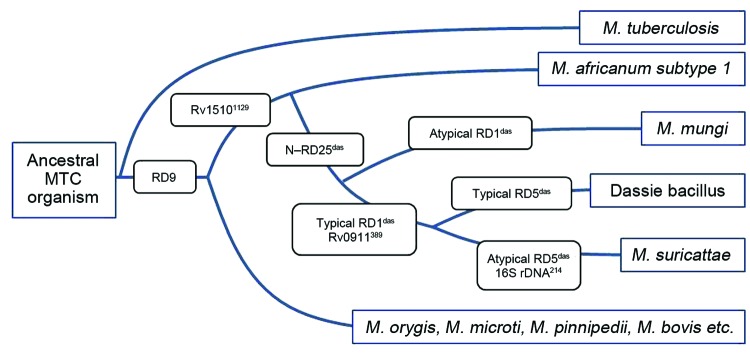
Phylogeny of the *Mycobacterium tuberculosis* complex (MTC) detailing relevant genetic regions of difference (RDs) and single-nucleotide differences that distinguish between *M. africanum* subtype 1 and the small African mammal–adapted members of these strains.

We genetically characterized the causative pathogen of meerkat TB as a novel MTC strain that has several genetic features typical of the dassie bacillus and *M. mungi* (Figure 2). However, this pathogen differs from the closely related dassie bacillus in its mycobacterial interspersed repetitive unit–variable number tandem repeats patterns (Table 2) by being a unique RD5^das^ variant and by containing 21 copies of the IS*6110* insertion element (Figure 1) (compared with 10–15 copies in the dassie bacillus) (*2*). This evidence of IS*6110* copy number expansion might indicate involvement of this insertion sequence in the occurrence of other genetic deletions in this strain, including those in the direct-repeat region. Notably, in addition to the novel SNP 16S rDNA^214^, the loss of the direct-repeat region spacers, which are routinely screened for by spoligotyping, distinguishes this strain from all other MTC members (*1*,*13*,*14*). As evidenced by their shared RDs and SNPs, the genetic homogeneity of multiple isolates of this distinctive strain suggests that it has undergone selective evolution, possibly through adaptation to its meerkat host (*1*). It is highly pathogenic in this species and seems to be substantially more virulent than the genetically similar dassie bacillus (*4*,*5*). As such, to distinguish this epidemiologically unique strain from other MTC members, we have named it *M. suricattae* after the host species from which it has been isolated.

The identification of this bacillus in Africa is further evidence that the early evolution of the animal-adapted MTC strains occurred on this continent. Strains derived from the early diversification of the RD9-deleted lineage include *M. africanum*, which has been almost exclusively isolated in West Africa (*2*); *M. mungi*, which was isolated from African mongooses (*6*); and *M. orygis* and the dassie bacillus, which have been isolated from animals mainly originating from this continent and the Middle East (*2*,*4*,*5*).

Of these strains, *M. africanum* subtype I and the dassie bacillus share a unique common progenitor (*2*); our study confirms the shared SNP Rv1510^1129^ as a genetic marker thereof (Figure 2). Given that *M. africanum* might have an unidentified West African animal host (*1*), it might be useful to consider that other members of this lineage have become established in highly gregarious small mammal hosts, including 2 mongoose species.

This study demonstrates that the occurrence of TB in the Kalahari meerkats might not be indicative of an external infectious source of *M. tuberculosis* or *M. bovis*, as has been reported (*6*,*8*). Rather, our findings suggest that the disease is caused by an indigenous MTC member, which we have named *M. suricattae*. Our limited sample set precludes a detailed analysis of the epidemiology of this pathogen; however, the identification of this strain and the characterization of several of its discriminatory genetic markers will be useful for future investigations of the ecology and evolution of the African animal–adapted members of the MTC.
